# Perceived Changes in Communicative Interaction in Atypical Parkinsonism

**DOI:** 10.5402/2011/256406

**Published:** 2011-04-13

**Authors:** Lena Hartelius, Johan Lindberg, Lena Petersson, Charlotta Saldert

**Affiliations:** Division of Speech and Language Pathology, Institute of Neuroscience and Physiology, Sahlgrenska Academy at the University of Gothenburg, Hälsovetarbacken Box 452, 405 30 Göteborg, Sweden

## Abstract

The aim of this study was to examine if atypical parkinsonism affects the communicative ability in conversational interaction. Fifteen persons close to individuals with atypical parkinsonism answered a questionnaire, “Assessment of Change in Communicative Interaction” (ACCI), estimating perceived change in interactive skills compared to before the onset of the disease. The study also examined if perceived change correlated with disease duration. The results showed that at group level, the participants experienced change in many aspects of conversational interaction, particularly regarding the affected person's speech, body communication, response latency, phrase length, word finding, and ability to make themselves understood. There was no correlation between perceived change and disease duration. In conclusion, results indicated that the communicative interaction of individuals with atypical parkinsonism is significantly affected and that information elicited from significant others can help define specific problem areas or foci of concern that need to be targeted in communicative intervention or at least considered in interaction with these persons.

## 1. Introduction

Changes in communication, brought about by neurological disorders, are most often defined and described in terms of the individual's impairments of speech and voice (dysarthria) or language (aphasia). Different aspects of speech and language can be measured and quantified using clinical tests and instrumental analyses. The individual's perception of degree of impairment and its impact can also be assessed, using qualitative interviews or self-report questionnaires. However, communication is per definition an interaction, a joint effort which makes the conversational partner a key player. This is true in all types of every-day conversations, but especially so when one of the interacting persons has a communicative impairment.

The necessary prerequisites in communicative interaction are intact sensori-motor processes (auditory and visual perception, voice and speech function, ability to gesture, change posture, etc.), linguistic ability (knowledge of the sound system, semantics, syntax, and discourse), and cognitive abilities (such as attention, memory, inference, executive function, affect, and the ability to infer mental states in others, i.e., theory of mind). These capacities all interact to form a person's pragmatic ability [1]. Consequently, the occurrence of any type of brain damage could have a negative impact on the ability to communicate in several different ways. 

There is a growing recognition that language impairments and pragmatic deficits occur in different neurodegenerative disorders such as Parkinson's disease. Affected abilities include interpretation of communicative intentions underlying verbal irony and lies, theory of mind, comprehension of metaphors, and the ability to use vocal cues to effectively infer a speaker's emotions and attitudes. The joint focus in these studies is the role of basal ganglia and frontostriatal systems in “complex” language processing [2–6]. Some studies also indicate that speakers might be unaware of the extent of their pragmatic communication problems [3].

Atypical parkinsonism, including progressive supranuclear palsy (PSP), multiple system atrophy (MSA), and the more infrequent corticobasal degeneration (CBD) are all associated with mixed types of dysarthria with varying severity [7–9]. In PSP, dysarthria is a common and often early and prominent sign [9, 10] and is, as opposed to the hypokinetic dysarthria in PD, characterized by signs of spasticity, for example, monotony, harsh voice, imprecise articulation, and slow rate. Other speech difficulties are often noted, such as palilalia, “stuttering” dysfluencies, and echolalia. Individuals with PSP often exhibit executive dysfunction such as difficulties with shifting mental set, problem solving, and abstract thinking [8]. In MSA, dysarthria is reported to be present in 100% of unselected cases [9] and is also mixed, with a predominance of ataxic signs such as slow speech with characteristic changes in stress patterns in MSA-C and a predominance of hypokinetic signs such as reduced stress, breathy hypophonic voice, and sometimes a rapid speech rate in MSA-P [9, 10]. CBD is characterized by multiple communication deficits, mixed dysarthria as well as apraxia of speech, and aphasia [9].

Despite the fact that communicative impairments are almost always occurring in atypical parkinsonism, studies describing changes in communicative interaction are basically nonexistent [11]. One case study of a woman with CBD included assessments of social language use and concluded that difficulties in topic management, turntaking, and making adequate contribution to the communication were increased [12]. Also, health-related quality of life has been measured in PSP [13] but failed to include items associated with communication, such as dysarthria, cognitive impairment, and lack of initiative.

How then should the communicative interaction be assessed and what are the relevant variables to measure? One line of investigation is in-depth analysis of video-documented real-life interactions (conversational analysis, CA [14]). However valuable, this procedure is time consuming and does not reflect particular changes, which might have occurred in the individuals being studied. Another type of research effort is represented by interviews and self-report questionnaires directed to the individual with the communicative impairment, asking about perceived changes. Given that persons with different types of brain damage tend to underestimate the extent of their communicative difficulties [15–17], another approach is to use significant others or carers as informants. One finding [16] shows that although it is true that persons with neurogenic communication disorders tended to rate their difficulties as less prominent than did the significant others, the correspondence between their ratings and the significant others' were statistically significant. Questionnaires directed to the significant other can also be considered a valid method, since the information is frequently based on long time experience of the person which means a possibility to make reasonably good estimate of changes in personality and behavior. It has also been found [18] that the agreement between information obtained from the carers and information drawn from analysis of video-recordings of conversations was reasonably high. 

Relevant variables to measure in communicative interaction, besides ability to comprehend and produce words, are the individuals' use of the conventions that participants in a conversation need to adapt to. One such convention is *turn-taking* patterns. There are several definitions of a conversational turn, and the speakers need to use, for example, syntactical and pragmatic signals in turntaking. Another convention is the *repair of problems in production or comprehension* in conversation. When a problem has occurred, self-initiated self-repair is preferred, that is, the person who produced the problematic utterance is also the one who changes it [19]. Nevertheless, help with the repair of an utterance is sometimes needed (other-initiated other-repair). Also, to make the conversational interaction functional, it is important to be able to use and to interpret communicative *body movements* and *feedback*. In addition, conventions on how to *introduce, change, and end different topics* of discussion need to be appropriately followed. 

A few questionnaires regarding changes in communicative interaction, directed to close others, have been used. Two of these were selected in the present study: Assessment of Change in Communicative Interaction (ACCI) [20] and La Trobe Communication Questionnaire (LCQ) [16, 21, 22]. The general aim of the present study was to investigate how significant others perceive that communication ability in conversational interaction has changed compared to before disease onset.

The specific questions asked were as follows.

1. Do the significant others perceive a change in communicative interaction after the onset of atypical Parkinsonism?
2. Does perceived change covary with time after onset?
3. Do the two selected questionnaires Assessment of Change in Communicative Interaction and La Trobe Communication Questionnaire correlate?

## 2. Methods

### 2.1. Participants

Participants in the study were significant others, related to persons who had been or were currently being investigated by the Movements Disorders Centre at the Neurology Clinic, Sahlgrenska University Hospital with atypical Parkinsonism being suspected. Exclusion criteria were communication disorders caused by stroke or any type of dementia, not associated with the disease. Thirty-one current patients were invited to participate in the study. Fifteen agreed to participate and selected a significant other, willing to answer two questionnaires (see Table 1 for a description of the patients).

Six women and 9 men participated in the study, age varying between 48 and 78 years. Twelve patients had multiple system atrophy (MSA) and 2 patients had progressive supranuclear palsy (PSP). One patient had not received a diagnosis but was considered probable PSP. Time after onset, according to significant others' assessment, varied between 2 and 11 years. 

The significant other was most often the patient's spouse, in other cases child, sibling, or close friend. The group of significant others had a mean age of 59 years, varying between 21 and 78, and 3 of them reported some kind of hearing difficulty. All patients and their significant others gave their written informed consent.

### 2.2. Questionnaires Regarding Changes in Conversational Interaction

To ensure concurrent validity, two different questionnaires regarding perceived changes in conversational interaction were used. Both questionnaires are directed to significant others. The first was “Assessment of Change in Communicative Interaction,” ACCI [20], an adaptation of a tool used for structured interviews with significant others, Conversational Analysis Profile for People with Cognitive Impairment, CAPPCI [23]. ACCI has been used in studies of individuals with subtle language problems due to stroke [20] and of individuals with Huntington's disease [24, 25]. The revised version of ACCI consists of 36 questions (see Appendix 1 in Supplementary Material available online at doi 10.5402/2011/256406) covering different areas of communication, such as (a) basic language ability, (b) turntaking, (c) topic management, (d) repair, (e) complex language comprehension, (f) attention and memory, (g) voice and speech (h) body communication, and (i) feedback. The significant other marks the frequency of occurrence of a total of 36 different behaviors on a five-point Likert scale. The five-scale steps are *Very often*; *Often*; *Occasionally*; *Rarely*; *Never*. The significant other reports the frequency of occurrence as they perceived it before disease onset and then uses the same scale to report the frequency of occurrence nowadays.

The second questionnaire was “La Trobe Communication Questionnaire”, LCQ [16, 21, 22], translated by permission of the authors and according to translation principles outlined by Wild et al. [26]. LCQ is designed for individuals with traumatic brain injury and covers communication abilities in conversation. The questionnaire comprises 30 questions (see Appendix 2 in Supplementary Material). For each question, the significant other reports how often a certain behavior (such as “leave out important details”) occurs, on a four point scale: (1) Never or Rarely, (2) Sometimes, (3) Often, and (4) Usually or Always. For every item, the significant other also reported whether there had been a change of the behavior after onset by choosing one of three alternatives, (+) Happens more, (0) No change, and (−) Happens less.

ACCI covers more aspects of communication (such as body communication and feedback) than LCQ does and also gives an indication of how large the communicative change is perceived to be, not only if it has occurred or not. However, LCQ has so far been more widely used, and its reliability and validity has been evaluated [21].

### 2.3. Procedure

A letter of information, both questionnaires, informed consent forms for the patient and the significant other, and a return envelope was sent to all patients (*n* = 31). The patients were asked to select a significant other who had known them before onset of the disease and who were willing to answer the questionnaires.

### 2.4. Statistical Analysis

For all statistical analyses, SPSS (version 15.0) was used. To avoid type 1 error, the *P* value was set at  .01. Results are mainly descriptive, and only nonparametric tests were used. Covariation between perceived changes in conversational interaction and time after onset as well as correlations between the two questionnaires was investigated using Kendall's tau(b), a nonparametric test suitable for analysis of a limited set of ordinal scale, not normally distributed data with a number of “tied ranks”, that is, several participants with the same results.

## 3. Results

Results are reported below, as answers to each of the three specific research questions.

**(1) Do the Significant Others Perceive a Change in Communicative Interaction after the Onset of Atypical Parkinsonism?**

**Figure 1:**
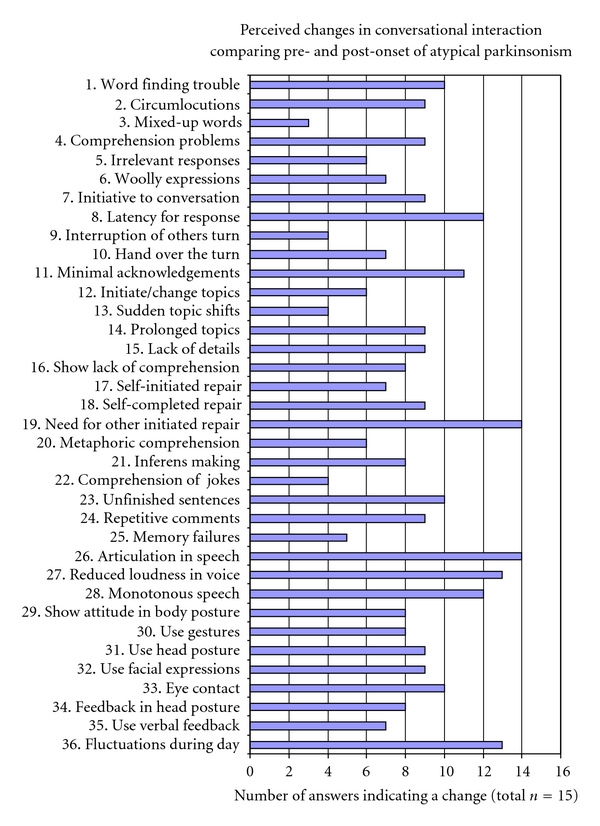
Number of participants (total *n* = 15) who answered that they perceive a change in their significant other since the onset of atypical Parkinsonism. The figure comprises all questions included in the Assessment of Change in Conversational Interaction (ACCI).

Figure 1 describes how many of the participants who perceived a change (corresponding to at least one scale step) in their significant other since the onset of atypical parkinsonism. As many as 14 of 15 participants perceived changes in clarity of articulation and changes in the ability to make oneself understood without help from significant others. Thirteen of 15 participants perceived changes in the ability to speak loud and had noticed that communication varied during the day. Changes in response latency, monotonous speech, and short answers were perceived by 12 and 11, respectively, of the 15 participants. Ten participants perceived changes with regard to eye contact, word finding, and the frequency of unfinished sentences (as a sign of attentional lapses).

A considerable variation was found, both in terms of *degree* of perceived change and the *number of questions* indicated by each participant reflecting perceived change. Degree of perceived change was defined as median-scale step change within each area of ACCI. In the area of “voice and speech”, a higher degree of change was noted, with a mean value of three-scale step's change. The areas “turn-taking”, “repair”, “attention and memory”, and “body communication” showed a median change of one-scale step. Least degree of change was found in the areas of “basic language ability”, “topic management” and “feedback,” and “complex language comprehension”. The variation within each area was between 0- and 4- scale steps. Each of the participants also noticed changes to different extents, reflected in the number of questions where they had noticed a change in their significant other. One participant only noticed changes in one of the questions, while another had noticed changes in 33 of the 36 questions. Median was 21 questions, mean 20.4 (S.D. 8.9). Included in the present study were 2 individuals with definite PSP and 12 with MSA. Median for the total number of questions indicating a change on ACCI was for the small group of PSP patients 32 and for the MSA group 19. The groups are too small and uneven in number to allow for statistical comparison, but the median was higher for the persons with PSP compared to MSA in all areas except “attention and memory” and “speech and voice.”

**(2) Does Perceived Change Covary with Time after Onset?**

Kendall's tau(b) does not show any statistically significant correlation between time after onset and the total number of questions in ACCI where the participants perceive a change, *τ* = −0.04, *P* = .84.

**(3) Do the Two Selected Questionnaires Assessment of Change in Conversation Interaction and La Trobe Communication Questionnaire Correlate?**

**Figure 2:**
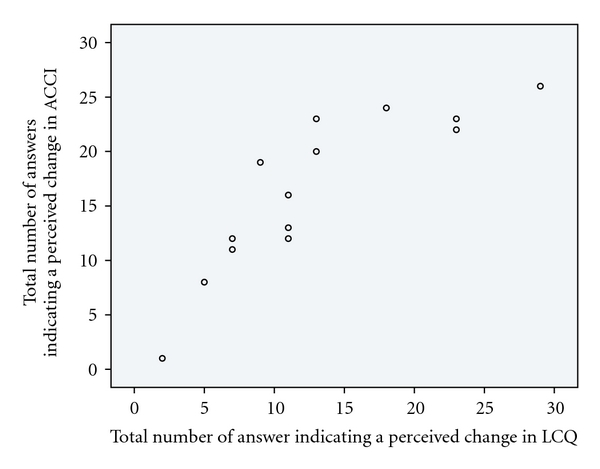
Co-variation between the total number of answers, based on 14 participants, indicating perceived change in Assessment of Change in Conversational Interaction (ACCI), maximum 28 answers and La Trobe Communication Questionnaire (LCQ), maximum 29 answers. Note that questions about body communication and feedback have been excluded here.

A strong, positive linear covariation was found between the total number of answers indicating a perceived change in ACCI and LCQ, see Figure 2. (Questions covering nonverbal communication and turntaking have been excluded, since there is only one single question in LCQ pertaining to eye contact.) The correlation is statistically significant (Kendall's tau(b) *τ* = 0.82, *P* < .01).

## 4. Discussion

In summary, change was perceived in all aspects of communicative interaction, as reflected in the ACCI. The most frequently reported problems were articulation in speech, being understood without aid from the communication partner, being too quiet, monotony of speech, variability (during the day), latency of response, short answers, finding words, unfinished sentences, and lack of eye contact. The perceived changes were not related to time after onset. 

Obviously, the investigated group represents a small and heterogeneous sample of individuals with atypical parkinsonism, which means that the results need to be interpreted with caution. The changes were indeed perceived to varying degrees, reflected by the number of answers indicating change. This can be explained by the small and heterogeneous group and the large individual differences in the disease symptoms of this group. Also, the results were based on the subjective ratings from significant others, not reflecting any “true” state of the person with atypical parkinsonism. However, it is worth noting, that the significant others of the persons with PSP reported larger perceived changes compared to the significant others of the persons with MSA. This could reflect the differing rate of progression between the two types of disease and also the higher prevalence of cognitive symptoms in PSP [7, 8].

A certain co-variation with time post-onset was expected but not found in the present study. One reason might be that there are large individual differences within the group of subjects with atypical parkinsonism, both in type and progression rate of different symptoms. Some individuals might have a rapid progression and severe symptoms early on, which is often the case in PSP, while others might have a slower progression [8].

The concurrent validity, measured as correlation between the two included instruments, proved to be satisfactory. Since the La Trobe Communication Questionnaire is validated, the positive correlation with ACCI strengthens the results of the present study. Concerning face validity, that is, the suitability of the questions included in ACCI for this specific population, it can be argued that ACCI includes a number of areas that are relevant, including body language, voice and speech, attention and memory, and turntaking. Also, difficulties found in a similar population, individuals with Parkinson's disease, such as deficient theory of mind, understanding of metaphors and irony, and other uses of complex language ability, are also covered by ACCI. 

The selected source of information in the present study was significant others. It has been shown that persons with Parkinson's disease have a tendency to overestimate their communication skills [15], and the same might be true, even to a higher degree, for individuals with atypical parkinsonism, because of rate of disease progression, cognitive involvement, and lack of insight. Significant others can also provide more reliable information about communicative function than health care professionals can, because they know the person better and interact with him/her more often [15, 18]. However, it is also possible that the significant other is so familiar with the communication and the communication partner that their interaction might function surprisingly well. The significant others might use conscious or subconscious strategies to compensate for difficulties that might reveal themselves more in interaction with other people. 

A measure of change, rather than studying actual communicative interaction, also has its advantages. Personality and style of communication is very individual, and a certain behaviour, which might be considered aberrant, needs not be a sign of illness but in fact a characteristic of the individual. Consequently, it is more informative to look at change within an individual rather than rate the characteristic. 

In the present study, the questionnaire was mailed to the participants, which might have influenced the rather low response rate (48%). In questionnaire studies, the response rate should be at least 50%. In clinical work, it would be preferable if the questionnaire was mailed to the significant other and subsequently reviewed together with him/her at a follow-up meeting to discuss the perceived changes and suitable strategies to meet the change. It might also be valuable for significant others to be invited to participate in a support group or structured conversation partner training, to increase understanding and decrease frustration in families affected by atypical parkinsonism.

## Supplementary Material

The Supplementary Material consists of two appendices. Appendix 1 comprises the 36 questions included in the version of Assessment of change in communicative interaction (ACCI) used in the present study. Appendix 2 consists of the 30 questions included in La Trobe Communication Questionnaire.

## Figures and Tables

**Table 1:** Patient characteristics.

	Age	Gender	Time post onset (years)	Diagnosis	Dysarthria severity	Augmentative communication devices	Ambulatory support	Memory difficulties according to significant other	Cognitive disability according to neuro-psychiatrist	Relationship to significant other
1	48	Male	4	MSA	Mild	No	Yes	Yes	No	Friend
2	64	Female	8	MSA	Mild	No	Yes	No	No	Spouse
3	59	Female	3	MSA	Mild	No	Yes	No	No	Spouse
4	62	Male	4	PSP	Mild/Moderate	No information	No information	Yes	No	Parent
5	65	Male	11	MSA	Severe	Yes	Yes	No	No	Spouse
6	59	Male	8	MSA	None	No	Yes	No	No	Parent
7	60	Male	9	MSA	Moderate/Severe	No	Yes	Yes	No	Spouse
8	76	Male	4	MSA	Mild/Moderate	No	Yes	No information	No	Spouse
9	67	Female	4	PSP?	Moderate	No	Yes	No	No	Sibling
10	67	Male	4	MSA	Mild	No	No	No	Yes	Spouse
11	67	Female	7	PSP	Moderate/Severe	No	Yes	Yes	Yes	Spouse
12	78	Male	6	MSA	Mild/Moderate	Yes	Yes	No	Yes	Spouse
13	56	Female	3	MSA	Mild	No	Yes	No	Yes	Spouse
14	64	Male	5	MSA	Mild	No	Yes	No	No	Spouse
15	58	Female	2	MSA	Mild	No	Yes	Yes	No	Parent
Mean	63.3		5.5							
Median	64		4							
