# Supplementary material for: Perceived Changes in Communicative Interaction in Atypical Parkinsonism
Source: ISRN Neurol. 2011 Apr 13;2011:256406. doi: 10.5402/2011/256406 (PMC3263542; doi:10.5402/2011/256406)
Supplement: Supplementary file 1 — The Supplementary Material consists of two appendices. Appendix 1 comprises the 36 questions included in the version of Assessment of change in communicative interaction (ACCI) used in the present study. Appendix 2 consists of the 30 questions included in La Trobe Communication Questionnaire. [file 256406.f1.pdf]

Appendix 1.Questions from the version of Assessment of change in communicative interaction (ACCI) used in the present study.

Each question is followed by two scales and the respondent is asked to circle the alternatives they feel best describe the frequency of the behaviour asked about both before the disease and nowadays:

Before the disease	**Very often**	**Often**	**Occasionally**	**Rarely**	**Never**
Nowadays	**Very often**	**Often**	**Occasionally**	**Rarely**	**Never**

A. Basic language ability
1. Does your close other struggle to find the appropriate word when s/he is talking?
2. *When* your close other can't find the appropriate words – does s/he tries to describe what s/he means in another way?
3. Does your close other use the wrong word for something? (For example, say 'cross' instead of 'crow' or 'apple' instead of 'pear'.)
4. Does your close other have difficulties understanding what you say to him/her?
5. If you ask a question to your close other – does s/he give you a response that is not a response to your question?
6. Does your close other use words like "him/her" and "there/here" without you knowing who or what s/he is referring to?

B. Turn-taking
7. Does your close other take initiative and starts up a conversation with you?
8. Can it be very long pauses or does s/he not respond at all when it is his/her turn to talk?
9. When you speak to each other, does s/he interrupts your turn?
10. When your close other speaks, does s/he have difficulties perceiving that you want to take the turn and say something?
11. Does your close other restrict her/his responses to minimal acknowledgements?

C. Topic management
12. In ordinary conversations we sometimes switch between different topics. Does your close other initiate a new topic or change the topic in your conversations?
13. When your close other introduces a new topic – do you feel the switch is interrupting or that it is difficult to see how the new topic fits in?
14. Does your close other continue talking about the same thing with you for a while?
15. Does your close other leave out important details when s/he is trying to tell you something?

D. Repair
16. Sometimes understanding others can be a problem for us all. When this happens to your close other, does s/he let you know that s/he hasn't really followed what you said? (This question is not about how often your close other does not understand what you are saying, but about how often s/he lets you know when it happens.)
17. Anybody can accidently mispronounce or choose the wrong word sometimes. *When* it happens to your close other, does s/he try to correct the mistakes by herself/himself?
18. If your close other tries to correct herself/himself – does s/he manage to correct the mistakes on her/his own?

19. Do you ever need to ask questions to your close other to be able to understand more exactly what s/he wants to say?

E. Complex language comprehension

20. Does your close other perceive figurative speech only at the literal, surface meaning and hence misunderstands?
21. Does your close other ‘read between the lines’ to understand what people really mean with what they say? (For example, if someone would say “it is rather cold in here”, would your close other understand that the person probably wants you to close the open window?)
22. Does your close other ever miss the point of jokes?

F. Attention and memory

23. Does your close other stop speaking in the middle of a sentence and leave it unfinished as if s/he has lost her/his thread or been distracted?
24. Does your close other ask you the same question or make the same remarks over and over again?
25. When you talk together about family, friends or events, does s/he forget who they are or what has happened?

G. Voice and speech

26. Does your close other have difficulty pronouncing the words clearly s/he speaks?
27. Does your close other talk so softly that you are unable to hear what s/he says?
28. Does the speech of your close other sound flat or monotonous?

H. Body communication

29. Can you read the body posture of your close other to tell what s/he really feels or thinks?
30. Does your close other use gestures when s/he talks about something, (for example showing size or how something looks like with the shape of her/his hands)?
31. Does your close other use head movements to express something, (for example, nod, shake the head or tilt the head)?
32. Does your close other express what s/he feels with facial expressions, (for example, knit her/his brows, smile or purse her/his mouth)?
33. Does your close other make eye contact with you when you talk together?

I. Feedback

34. Does your close other give you feed back on what you are saying with head movements, (for example, nods when agreeing with you, or shake her/his head when you are telling her/him about something that has been difficult for you)?
35. Does your close other give you feedback on what you are talking about by saying for example, ‘yes’, ‘no, or ‘mmm’ or make similar sounds, as you speak?
36. Think of a typical day. Does your close other’s communication abilities fluctuate during the day?

Appendix 2.Questions from La Trobe Communication Questionnaire

Each question is associate with two indicators, frequency and change, and the responden is asked to circle the alternative they feel best describe the behaviour of the close other and the perceived change. For example:

When talking to other does ________________:

	FREQUENCY	CHANGE
1. Leave out important details?	1 2 3 4	+ 0 -

When talking to other, does ______________(name)____________:

1. Leave out important details?
2. Use a lot of vague or empty words such as “you know what I mean” instead of the right word?
3. Go over and over the same ground in conversation?
4. Switch to a different topic of conversation too quickly?
5. Need a long time to think before answering the other person?
6. Find it hard to look at the other speaker?
7. Have difficulty thinking of the particular word he/she wants?
8. Speak too slowly?
9. Say or do things others might consider rude or embarrassing?
10. Hesitate, pause and/or repeat him/herself?
11. Know when to talk and when to listen?
12. Get side-tracked by irrelevant parts of conversations?
13. Find it difficult to follow group conversations?
14. Need the other person to repeat what they have said before being able to answer?
15. Give people information that is not correct?
16. Make a few false starts before getting his/her message across?
17. Have trouble using tone of voice to get the message across?
18. Have difficulty getting conversations started?
19. Keep track of the main details of conversations?
20. Give answers that are not connected to the questions asked?
21. Find it easy to change his/her speech style (e.g. tone of voice, choice of words) according to the situation he/she is in?
22. Speak too quickly?
23. Put ideas together in a logical way?
24. Allow people to assume the wrong impressions from his/her conversations?
25. Carry on talking about things for too long in his/her conversations?
26. Have difficulty thinking of things to say to keep conversations going?
27. Answer without taking time to think about what the other person has said?
28. Give information that is completely accurate?
29. Lose track of conversations in noisy places?
30. Have difficulty bringing conversations to a close?
